# Autism prevalence in China is comparable to Western prevalence

**DOI:** 10.1186/s13229-018-0246-0

**Published:** 2019-02-28

**Authors:** Xiang Sun, Carrie Allison, Liping Wei, Fiona E. Matthews, Bonnie Auyeung, Yu Yu Wu, Sian Griffiths, Jie Zhang, Simon Baron-Cohen, Carol Brayne

**Affiliations:** 1 Cambridge Institute of Public Health, Department of Public Health and Primary Care, University of Cambridge, Forvie Site, Robinson Way, Cambridge, CB2 0SR UK; 2 Autism Research Centre, Department of Psychiatry, University of Cambridge, Douglas House, 18b Trumpington Road, Cambridge, CB2 2AH UK; 3 Department of Psychology, The Chinese University of Hong Kong, 3rd Floor, Sino Building, Shatin, NT Hong Kong; 4 Centre for Bioinformatics, School of Life Sciences, Peking University, Beijing, 100871 People’s Republic of China; 5 National Institute of Biological Sciences, Beijing, China; 6 Institute of Health and Society, Newcastle University, Newcastle, NE4 5PL UK; 7 Department of Psychology, School of Philosophy, Psychology and Language Sciences, University of Edinburgh, Edinburgh, EH8 9JZ UK; 8 YuNing Psychiatry Clinic, YuNing Growing With You Mental Health Centre, Taipei, Taiwan; 9 The Jockey Club School of Public Health and Primary Care, The Chinese University of Hong Kong, Shatin, NT Hong Kong

**Keywords:** Autism, Screening, Diagnosis, Prevalence, Children, China

## Abstract

**Background:**

Autism prevalence in the West is approximately 1% of school age children. Autism prevalence in China has been reported to be lower than in the West. This is likely due to at least two reasons: (1) most studies in China only included the special school population, overlooking the mainstream school population; and (2) most studies in China have not used contemporary screening and diagnostic methods. To address this, we tested total autism prevalence (mainstream and special schools) in Jilin City, and mainstream school autism prevalence in Jiamusi and Shenzhen cities.

**Methods:**

The study included a three-step process: (1) screening; (2) clinical assessment of ‘screen positives’ plus controls; and (3) research diagnostic assessment of those meeting clinical threshold for concerns at step 2. Prevalence estimates per 10,000 children aged 6–10 years old were weighted for study design using diagnostic criteria applied at the research assessment stage.

**Results:**

In Jilin City, 77 cases of autism were identified from a total population of 7258, equating to a prevalence of 108 per 10,000 (95% confidence interval (CI) 89, 130). In Shenzhen City: 21,420 children were screened and 35 cases of autism were identified, resulting in a mainstream prevalence of 42 per 10,000 (95% CI 20–89). In Jiamusi City, 16,358 children were screened, with 10 autism cases being identified, with a mainstream prevalence of 19 per 10,000 (95% CI 10–38).

**Conclusions:**

Results from Jilin City, where both mainstream and special school data were available, revealed a similar prevalence of autism in China to the West, at around 1%. Results from Shenzhen and Jiamusi cities, where only mainstream data were available, prevalence is also in line with Western estimates. In all three cities, new cases of autism were identified by the study in mainstream schools, reflecting current under-diagnosis. Non-significant variation across different cities is seen indicating the need to explore potential variation of autism across diverse Chinese regions with large sample sizes to achieve a fully robust national picture.

**Electronic supplementary material:**

The online version of this article (10.1186/s13229-018-0246-0) contains supplementary material, which is available to authorized users.

## Introduction

Autism spectrum conditions (henceforth autism) are characterised by impairments in social interaction and communication, alongside the presence of unusually repetitive behaviour and narrow interests, difficulties adjusting to unexpected change, and sensory hyper-sensitivity [1]. The autism spectrum includes marked heterogeneity in intelligence and language development. Population-based epidemiological studies in the West have reported increases in the prevalence of autism over time, ranging from 30.8 per 10,000 in 2000 [2], to 157 per 10,000 in 2009 [3] to 169 per 10,000 in 2018 [4].

Autism was first described in Western cultures, and only later recognised in Asian countries [5]. Understanding the prevalence of autism in Asian countries is important because of its relevance to service planning, and for our understanding of the genetic and environmental contributing factors of autism in diverse populations. In a South Korean study in 2011, autism population prevalence was reported to be 264 per 10,000 and 189 per 10,000 (95% CI 143–236) in mainstream schools [6]. This high estimate may have resulted from the particular screening and assessment instruments used [7]. In China, most prevalence studies of autism have only focused on one subtype, that is, children with autism who have intellectual disability, omitting children without intellectual disability, including those who may previously have been diagnosed with Asperger syndrome (Appendix 1). This is despite the fact that 75% of the autism spectrum does not have intellectual disability [4, 8]. A systematic review reported the prevalence of autism in Mainland China, Hong Kong, and Taiwan to be 26.6 per 10,000 [8]. A recent review reported the pooled prevalence of autism in China was 39.23 per 10,000 [9], which is significantly lower than estimates from the West, suggesting the possibility of under-diagnosis. The current study tests two possible reasons for this lower autism prevalence in China, namely, that in many studies (1) autism in mainstream schools was overlooked; and (2) contemporary screening and diagnostic methods were not used.

This study focuses on mainstream schools because the *Sui Ban Jiu Du* policy encourages children with disabilities to attend mainstream school [10], although in practice children with moderate to severe autism are rarely enrolled in mainstream schools [11]. We have previously reported an autism prevalence estimate in mainstream primary schools in Beijing of 119 per 10,000 (95% CI 53, 265) [14] which is in line with Western autism prevalence estimates. We used a validated screening tool, the Childhood Autism Spectrum Test (CAST), and two research diagnostic assessment tools, the Autism Diagnostic Observation Schedule (ADOS) [12], and the Autism Diagnostic Interview-Revised (ADI-R) [13]. Most of the children identified in this study using these contemporary screening and diagnostic measures had not previously received an autism diagnosis, confirming under-diagnosis of autism in mainstream schools.

**Fig. 1:**
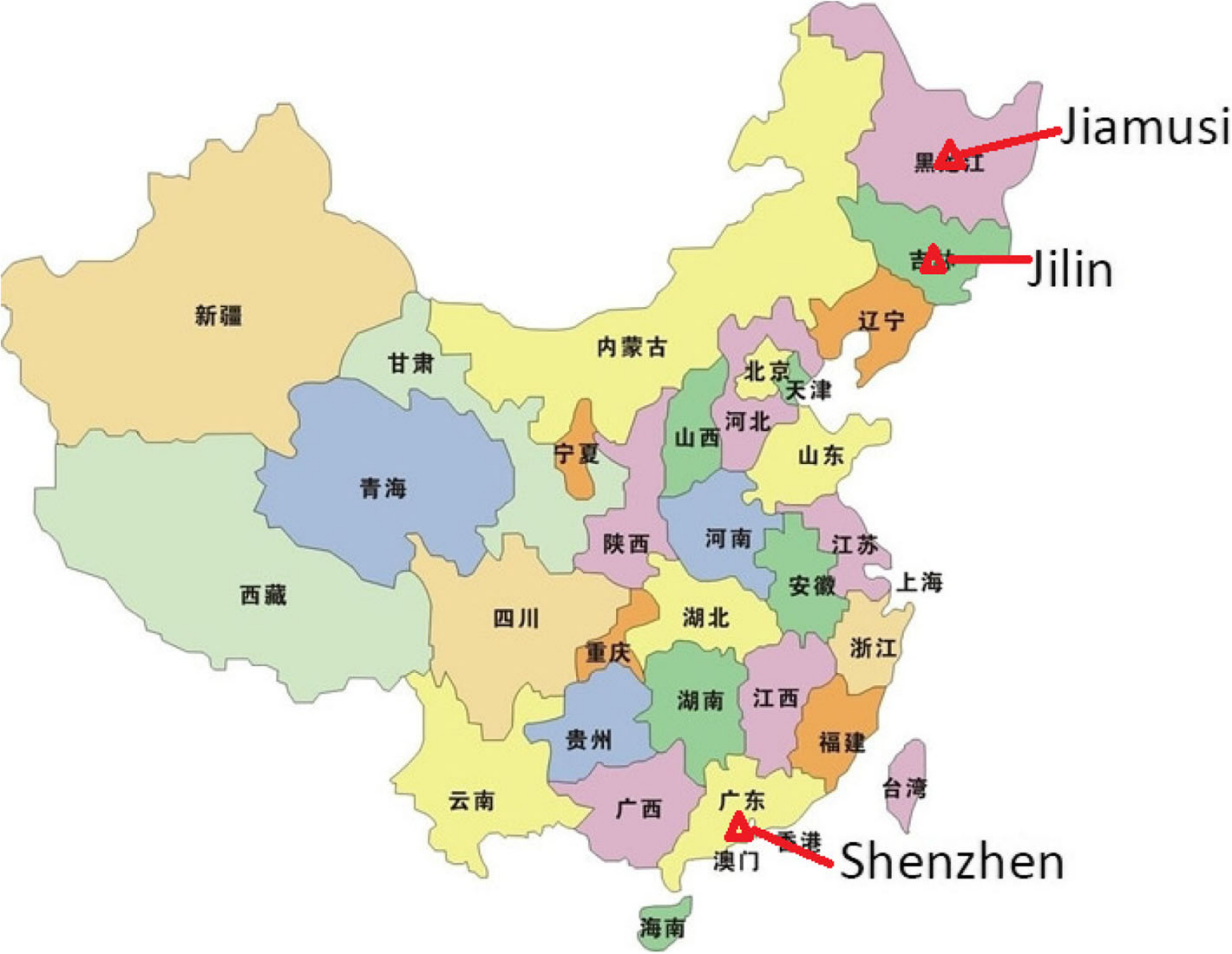
Location of three cities in China studied in phase I of the China SCORE study

Here, we report initial data from the China SCORE (Social Communication Research and Epidemiology) study, which aims to compare autism prevalence in China with estimates from the West. First, we report total autism prevalence (mainstream and special schools) in Jilin City. Second, we report autism prevalence in mainstream schools only in Shenzhen and Jiamusi cities. We replicate just the mainstream prevalence in these two cities, because mainstream prevalence has been so neglected in previous studies in China (see Fig. 1).

## Methods

### Screening instrument

The CAST is a 37-item parent-completed questionnaire, of which 31 items are scored. Thus, the total score ranges from 0 to 31. It was originally validated in the UK [15–19]. It has also been used in genetic studies [20]. A Mandarin translation of the CAST has been used to determine that using a cut-off of 15 (sensitivity = 84%, specificity = 96%), the Mandarin CAST can be used as a screening instrument in population-based epidemiological research for autism in China [21].

### Population sample

We screened children attending mainstream primary schools in the three cities. By definition, they are not the children reported in previous prevalence studies of children with autism in China attending special education schools. The inclusion criteria for selecting each of the three cities were as follows: the city should have (1) a median economic level (neither extremely affluent nor extremely poor); (2) an approximate sample size of 20,000 children aged 6 to 10 years old (children should not have had their 11th birthday at the time of the study); (3) a professional capacity to participate; (4) a median population mobility. These data were obtained from the National Statistics Bureau (http://www.stats.gov.cn/english/). Children in Mainland China are only admitted to primary school after their sixth birthday. In mainstream schools, grades 1 to 4 represent the age group 6 to 10 years old. Three cities met these criteria.

In Jilin City, all mainstream schools (*N* = 14) and special schools from special education (*N* = 3) in Fengman District for children aged 6–10 were invited, and all agreed to participate in the study. The local residence records were assessed and tracked for children with an autism diagnosis who did not attend school in the target age range.

In Shenzhen City, there are six administrative districts. Longgang District was selected for this study and out of the 11 communities in this district, 7 agreed to participate. There are 45 mainstream schools within these 7 communities, all of which agreed to participate. All caregivers of children in grades 1 to 4 were invited to participate in December 2013, representing a population of 20,553.

In Heilongjiang province, Jiamusi City was selected. Twenty-seven mainstream primary schools in Jiamusi City were invited, and all agreed to participate, representing a population of 16,358 in grades 1 to 4 in Jiamusi City. No special schools in Shenzhen and Jiamusi cities are included in this report.

### Case identification (diagnostic methods)

Most Western autism prevalence studies (e.g. US and UK) conducted standardized diagnostic instruments (ADOS and ADI-R) together with clinical diagnosis. In previous Chinese prevalence studies, clinical diagnosis was adopted as the gold standard. In order to be comparable with these existing studies, both diagnostic approaches were adopted in this study. This was in part to make sure that we captured all possible cases using a standardized protocol across each city. The study was conducted in 3 steps:

#### Step 1: Screening

In all three cities in December 2013, the principals of the participating schools were contacted and a screening package was provided to the head teachers of classes in grades 1 to 5 to distribute via students for their parents to complete at home. Screening data collection and data entry in Shenzhen City took 1 month to complete. In Jiamusi, it took over 3 months to complete. One percent of the data were double entered to quality control the data entry. A report was provided for all CAST items which were wrongly entered (enter value other than 0 or 1 or missing). If questionnaires missed more than five scorable items, parents were contacted and asked to complete the CAST a second time. A randomly selected 1% of questionnaires were checked for consistency in data entry compared to the hard copy questionnaires. Data entry was repeated if there was less than 95% agreement between the hard copy and the electronic data entry.

#### Step 2: Clinical assessment

Following quality checks on the data entry, all children in the high-score group (CAST ≥15) were invited for a further assessment. Five percent of children in the borderline group (CAST 12–14) were randomly selected and invited for a further assessment. If participation in the borderline group was less than 50%, another random sample of 10% of this borderline group was invited. In order to maximise the identification of children with autism in the low-score group (CAST ≤11), the school psychologists were asked to select children for a further assessment if they had previous concerns about the child’s behaviour. The clinical assessment team was blind to the screening status of all the children during the assessment.

A maximum of 30 children from each school in the low-score group were invited by the school psychologists. This was due to the variability of uptake for the clinical assessment in the low-score group and the variable resource availability in different regions. Thus, each grade could have invited a maximum of eight children. Participating families were offered incentives (100 RMB) for taking part in the clinical assessment. DSM-IV-TR and DSM-5 were the clinical diagnostic criteria used in this study by the child psychiatrists. The clinical assessment team comprised child psychiatrists with expertise in autism, who were approved by the China Disabled Persons’ Federation (CDPF).

Agreement between clinicians of diagnostic outcome was established in three stages. First, CDPF in each city recommended child psychiatrists with expertise in diagnosing children with autism. Second, a core clinical expert team developed a diagnostic agreement test for child psychiatrists. We invited six child psychiatrists considered to be the most experienced in the diagnosis of autism in Mainland China to develop a test of diagnostic agreement. Ten children aged six to 10 years old in Shenzhen in April 2014 were selected, comprising *N* = 8 children with autism, *N* = 1 child with other developmental difficulties not related to autism and *N* = 1 neurotypical child. They were initially assessed by the first author and their diagnoses confirmed using the ADOS and ADI-R.

Second, they were randomly assigned to, and assessed independently by, the six child psychiatrists. When one child psychiatrist conducted a clinical interview, another psychiatrist observed. The diagnoses of the 10 children were discussed and agreed between the first author and the six child psychiatrists. This process was video recorded and edited to produce a training DVD for other child psychiatrists to use to establish diagnostic agreement.

Finally, the clinical experts completed the diagnostic agreement test and joined the clinical assessment team if s/he achieved diagnostic agreement of 80% or above.

This standardised clinical diagnostic protocol was established in order to ensure reliability of the clinical diagnosis across each region. The clinical assessments consisted of three parts: a face-to-face interview with parents; direct observation and communication with the child using toys; and questions relating to the child’s general development, together with completing the diagnostic forms. Two standardised diagnostic forms were provided to each child psychiatrist. One was the DSM-IV-TR criteria and the other was DSM-5 criteria. A third document was a supplementary question list based on examples from DSM-5 and DSM-IV-TR items. These questions were drawn from the ADI-R. This document was provided as a guide for possible questions to enquire about, but was not compulsory for the psychiatrists to use. The final clinical diagnosis was made in three categories: autism, suspected autism, and not-autism. The psychiatrist was asked to provide notes on the diagnostic form if they believed the child to have a developmental condition that was not related to autism.

#### Step 3: Research diagnostic assessments

After the clinical assessment (step 2), children whose clinical diagnostic outcome was autism or suspected of autism were invited for a research diagnostic assessment using the ADOS, the ADI-R. A measure of IQ was obtained using the Raven Progress Matrix (RPM) [16]. The Chinese version of the RPM was used, which is a validated measure and applicable to individuals from the age of 5 to 75 in Mainland China [23]. Ten children in Shenzhen City whose clinical diagnostic outcome was not autism were also randomly selected for a research diagnostic assessment to confirm whether there were potential cases of autism being missed by the clinical diagnostic team. ADOS and the ADI-R were conducted by researchers who had obtained research reliability prior to the study. Autism cases were defined using a consensus case definition.

If the child scored above the cut-offs for autism or autism spectrum on both the ADOS and the ADI-R, a research diagnosis of autism was made. If the child scored on both the ADOS and the ADI-R, or if the child scored above the diagnostic algorithm threshold on either the ADOS or the ADI-R, he or she was referred to a third clinical child psychiatrist in the clinical team. The third child psychiatrist used all information available from the assessment together with clinical judgement and consultation with DSM-IV-TR/DSM-5 diagnostic criteria. If there was any disagreement between the clinical diagnosis and research diagnosis (using the ADOS and ADI-R), a final diagnosis was made by consensus by the two child psychiatrists (using DSM-IV-TR and DSM-5) and the research assessment examiner (using the ADOS and ADI-R). In Jilin City, children with autism who were tracked from the local residence records were reassessed by child psychiatrists using the ADOS and ADI-R. The final diagnosis of these children was made by consensus diagnosis. More detailed methods are presented in Additional file 1.

### Data analysis

The distribution of CAST scores was examined using the skewness-kurtosis test. The impact of non-participation in the assessment phase was examined by comparing the characteristics of participants who took part in the assessment with those who were invited but refused to participate. The randomly selected sample based on the random number table (5%) from the borderline group (12–14) and the low-score group (≤ 11) who participated in the further assessment were compared with those who were not invited and who refused to participate separately. Differences between children who participated in the clinical diagnosis were examined in two steps.

For high and low CAST score groups, differences between children who did and did not complete the clinical assessments were examined. For the borderline CAST score group, children were divided into three groups: (1) children who were invited and completed the clinical assessments; (2) children who were invited but did not complete the assessments; (3) children who were not invited for a clinical assessment. The skewness-kurtosis test was used to examine the normality of the score distribution.

For ordered categorical or continuous and non-normally distributed variables, the Mann-Whitney test was used to compare medians for two groups, while the Kruskal-Wallis *H* test was used for multiple groups. Unpaired *t* tests and one-way analyses of variance (ANOVA) were adopted to compare means for normally distributed continuous variables, and the chi-square tests were used to examine differences in proportions for nominal or unordered categorical variables. Whenever the numbers were small, a Fishers’ exact test was used. All the analyses were conducted using STATA 14.0.

For all missing values on the CAST, a score of 0 was given to generate a minimum score. A score of 1 was given to generate a maximum score. A middle score was generated using the equation: (minimum + maximum)/2. Inverse probability weighting using sampling weights was applied to adjust the prevalence estimates for the known non-response to the invitation for assessment within each sampling score group [2, 3]. This strategy was used because of the two-phase sampling strategy. The inverse probability was the empirical weight generated according to the response to the screen and to the participation rate in the further assessment phase. A raw prevalence estimate was generated by first using the inverse probability weighting. Non-response weights were calculated between those invited for a clinical assessment and those that did not take part, allowing for differences in response for age, sex, education of the parents, and screening score (including missing item information). Any further non-response from the clinical assessment to the research assessment was also adjusted for using inverse probability weighting methods. Missing data were imputed, and an adjusted prevalence was provided after adjusting for age, sex, and the non-response differences. 95% confidence intervals were calculated accordingly by applying the weighted count.

## Results

### Jilin City: prevalence estimate

**Fig. 2:**
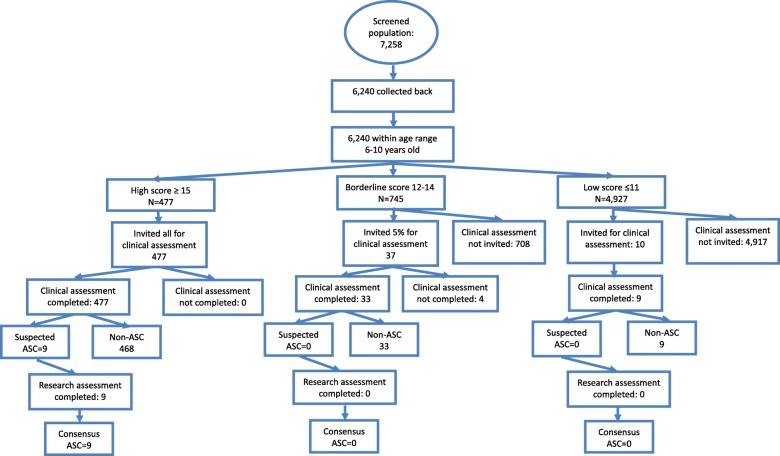
Flowchart of Jilin City

The characteristics of the parents are shown in Table 1. The study population in mainstream schools was 7167 (Fig. 2). Of these, 3282 (45.8%) were boys, 2883 (40.2%) were girls, for the rest 1002 (14.0%), and gender information was missing. The mean age of the sample was 8.5 years old (SD = 1.1). Occupational and educational level of the parents was also collected and divided into five categories [4].

**Table 1:** Characteristics of parents in three cities

Characteristics	Category	Shenzhen City		Jiamusi City		Jilin City		
		Number	(%)	Number	(%)	Category	Number	(%)
Mother’s education	Junior high school	6980	33.6	6413	41.5	Junior high school	1522	23.7
	High school	8316	40.0	5211	33.7	High school	2009	31.3
	College	4872	23.4	3373	21.8	College	2061	32.1
	Graduate school	154	0.7	252	1.6	Graduate	276	4.3
	Missing	479	2.3	219	1.4	Missing	552	8.6
Father’s education	Junior high school	4951	23.8	6092	39.4	Junior high school	1104	17.2
	High school	8208	39.5	5337	34.5	High school	1900	29.6
	College	6604	31.8	3445	22.3	College	2408	37.5
	Graduate school	374	1.8	310	2.0	Master or higher	372	5.8
	Missing	665	3.2	284	1.8	Missing	636	9.9
Mother’s occupation	Government officer	89	0.4	438	2.8	Worker or farmer	1682	26.2
	Company clerk	6173	29.7	1858	12.0	Clerk	1438	22.4
						Technical staff	1393	21.7
	Industry	1475	7.1	1806	11.7	Manager	161	2.5
	Self-employed	5461	26.3	4655	30.1	Own-business	995	15.5
	Worker	1578	7.6	1354	8.6	Missing	751	11.7
	Student	32	0.2	7	0.1			
	Farmer	937	4.5	2032	13.1			
	Unemployed	4002	24.1	3109	20.1			
	Missing	55	0.3	209	1.4			
Father’s occupation	Government officer	293	1.4	782	5.1	Worker or farmer	1367	21.3
	Company clerk	7518	36.1	1628	10.5	Clerk	1830	28.5
	Industry	1836	8.8	1634	10.6	Technical staff	1284	20.0
	Self-employed	7617	36.6	5022	32.5	Manager	276	4.3
	Worker	1690	8.1	2771	17.9	Own-business	1021	15.9
						Missing	642	10.0
	Student	10	0.1	5	0.0			
	Farmer	588	2.8	2071	13.4			
	Unemployed	1199	5.8	1305	8.4			
	Missing	51	0.3	250	1.6			
Mother’s age	≤ 24	61	0.3	36	0.2			
	25–30	2552	12.3	1981	12.8			
	31–34	11,921	57.3	8603	55.6			
	35–40	4816	23.2	945	6.1			
	≥ 40	410	2.0	2	0.0			
	Missing	1042	5.0	3901	25.2			
Father’s age	≤ 24	32	0.2	25	0.2			
	25–30	677	3.3	769	5.0			
	31–34	10,468	50.3	10,229	66.1			
	35–40	7577	36.4	1589	10.3			
	≥ 40	985	4.7	2	0.0			
	Missing	1063	5.1	3854	18.5			
Income	≤ 1999	829	4.0	2699	17.5			
	2000–3999	3355	16.1	5252	34.0			
	4000–5999	3836	18.4	3880	25.1			
	6000–7999	3050	14.7	1704	11.0			
	8000–9999	2389	11.5	569	3.7			
	≥ 10,000	5399	26.0	569	3.7			
	Missing	1944	9.4	795	5.1

The median score on the CAST was 8 (IQR 5, 11; range 0, 23). Of the 6149 screened children from mainstream schools whose data were available for analysis (step 1), 477 (7.8%) were in the high-score group on the CAST (≥ 15), 745 (12.1%) were in the borderline group (12–14), and 4927 (80.1%) were in the low-score group (≤ 11). All 477 children in the high-score group completed the clinical assessment (step 2) (participation rate = 100%). In the borderline group, 33 children participated of the 37 invited (89%). Nine children of the 10 invited in the low-score group participated (90%). Seven children were judged at clinical assessment to have autism and two were judged to have suspected autism, all of whom were from the high-score group. These nine children were invited for a research diagnostic assessment (step 3), and they all received a research diagnosis of autism.

Thus, when adjusting for non-response, the prevalence of autism in mainstream primary schools was 14.6 per 10,000 in Jilin City. Mean IQ score for the nine children was 105. Using the same three-step design and methods, 68 additional children with autism were found from 91 children studied from other settings serving this geographical locality. These included 19 from special schools, 43 from private intervention centres, and 6 children from the community not attending school. These six children did not have the resources to attend any schools or intervention centres. Thus, the overall prevalence estimate for autism in Jilin City (total population prevalence) was 108.0 per 10,000 (or 1 in 92) (95% CI 87.0, 135.0).

### Shenzhen City: prevalence estimate

**Fig. 3:**
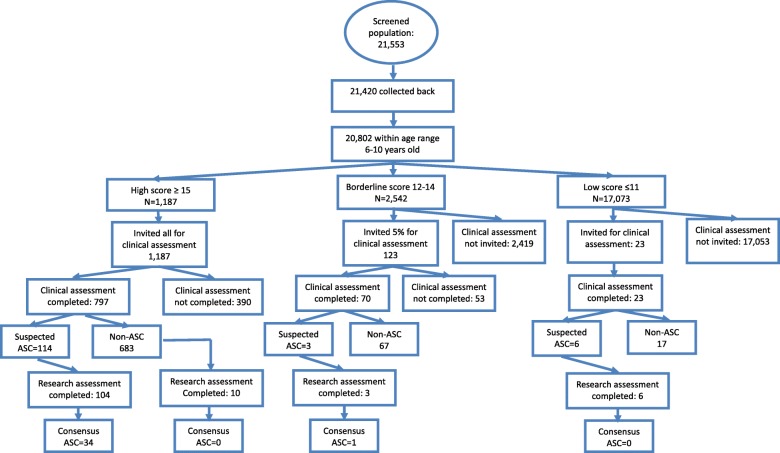
Mainstream-school screening in Shenzhen City

21,420 out of 21,553 (participation rate = 99.4%) screening questionnaires were completed and returned to the study team from 45 schools (Fig. 3). Of these, 11,878 (55.5%) were boys and 9312 (43.5%) were girls. None of these children had a diagnosis of autism before this study. For 230 (1.1%), gender was missing. Fifty-five children were excluded because they were younger than 6 years old and 86 were excluded because they were over 11 years old. Six were excluded because their year of birth was illegible. Of the 20,802 children (step 1) aged 6 to 10 (97.1%), 1187 (5.7%) were in the high-score group on the CAST (scoring ≥ 15), 2542 (12.2%) were in the borderline group (scoring 12–14), and 17,073 (82.1%) were in the low-score group.

In the high-score group, *N* = 1187 were invited for assessments, of whom 797 took part in the clinical assessment (step 2), with 114 referred for a research diagnostic assessment (step 3). One hundred four of these completed the research diagnostic assessment (91.2%), resulting in 34 children being diagnosed with autism.

In the borderline group, a random sample of 5% (123 students) were invited for a clinical assessment (step 2) and 70 completed (56.9%), with three referred for a research diagnostic assessment (step 3), all of whom completed. One child was diagnosed with autism at the research assessment.

In the low-score group, 23 children were identified by teachers with concerns about their behaviour and undertook the clinical assessment (step 2), of whom 6 were referred for and undertook the research diagnostic assessment (step 3). However, none of these six children met autism diagnostic criteria.

The 35 cases confirmed by the research diagnostic assessment equate to a prevalence of 42.3 per 10,000 children age 6 to 10 in mainstream education (95% CI 20.1–88.6) after adjusting for non-response (or 1 in 238). The prevalence estimate for boys was 38.8 (95% CI 26.5–56.7) and girls 44.9 (95% CI 10.1–196) per 10,000. Mean IQ score was 114 in these 35 children with autism. More detailed results of clinical and research diagnosis are in Tables 2 and 3.

**Table 2:** Clinical and research diagnosis across different CAST score groups in Shenzhen

Shenzhen City	Clinical diagnosis							Research diagnosis			
	Non-autism (%)	Autism or suspected autism (%)	ADHD	Developmental delay (%)	Not assessed (%)	Total (%)		Non-autism (%)	Autism (%)	Missing (%)	Total (%)
High score	670 (56.4)	114 (9.6)	12 (1.0)	1 (0.1)	390 (32.8)	1187 (100.0)		60 (57.7)	34 (32.7)	10 (9.6)	104 (100.0)
Borderline	67 (2.6)	3 (0.1)	0 (0.0)	0 (0.0)	2472 (97.3)	2542 (100.0)		2 (2.6)	1 (0.1)	0 (97.3)	3 (100.0)
Low	17 (0.1)	6 (0.0)	0 (0.0)	0 (0.0)	17050 (99.9)	17073 (100.0)		6 (0.1)	0 (0.04)	0 (99.9)	6 (100.0)
Total	754 (3.6)	123 (0.6)	12 (0.1)	1 (0.0)	19912 (95.7)	20802 (100.0)		87 (3.6)	35 (0.6)	0 (95.7)	113 (100.0)

**Table 3:** Clinical and research diagnosis across different CAST score groups in Jiamusi

Jiamusi City	Clinical diagnosis							Research diagnosis			
	Non-ASC (%)	ASC or suspected ASC (%)	ADHD	DD (%)	ID	Not assessed (%)	Total (%)	Non-autism (%)	Autism (%)	Missing (%)	Total (%)
High score	390 (43.3)	20 (2.2)	0 (1.0)	1 (0.1)	3 (0.3)	487 (54.1)	901 (100.0)	11 (55.0)	9 (45.0)	0 (0.0)	20 (100.0)
Borderline	143 (7.9)	1 (0.1)	0 (0.0)	0 (0.0)	0 (0.0)	1678 (92.1)	1822 (100.0)	1 (100.0)	0 (0.0)	0 (0.0)	1 (100.0)
Low	747 (5.8)	3 (0.0)	0 (0.0)	0 (0.0)	0 (0.0)	12190 (94.2)	12940 (100.0)	2 (66.7)	1 (33.3)	0 (0.0)	3 (100.0)
Total	1280 (8.2)	24 (0.2)	0 (0.0)	1 (0.0)	3 (0.0)	14355 (91.7)	15663 (100.0)	14 (58.3)	10 (41.7)	0 (0.0)	24 (100.0)

### Jiamusi City: prevalence estimate

**Fig. 4:**
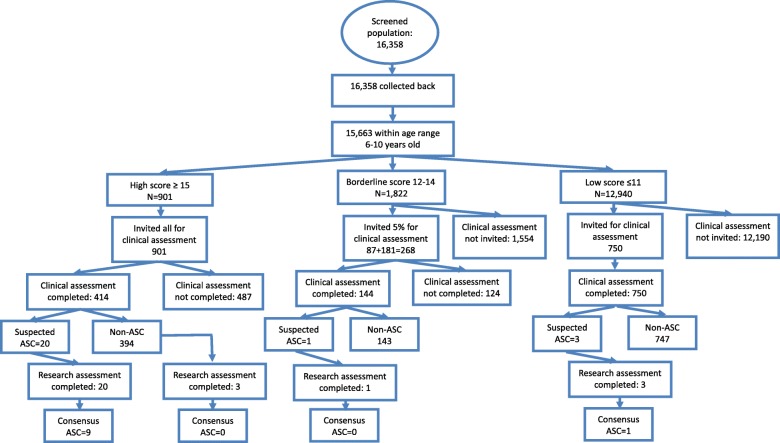
Flowchart of Jiamusi City

16,358 questionnaires were distributed to 27 schools with children aged 6 to 10 years old (Fig. 4). None of these children had a diagnosis of autism before this study. All were returned and available for analysis (participation rate = 100%). Of these, 8326 (50.9%) were boys and 7853 (48.0%) were girls. For 179 (1.1%), gender was missing. Age was within the correct range for 15,663 children (95.8%). The remaining children were excluded because they were younger than 6 years old (*n* = 195) or older than 11 years (*n* = 695), or their year of birth was illegible (*n* = 617). Of the 15,663 children within the age range (6–10), 901 (5.8%) fell in the high-score group (≥ 15), 1822 (11.6%) were in the borderline group (12–14) and 12,940 (82.6%) were in the low-score group (Appendix 1).

In the high-score group, 414 (45.9%) of the invited 901 took part in the clinical assessment (step 2), with 20 referred for and completing a research diagnostic assessment (100%) (step 3), among whom 9 received a diagnosis of autism.

In the borderline group, a random sample of 5% (*n* = 87) and a second sample of 10% (*n* = 181) were invited for a clinical assessment (step 2). One hundred forty-four of these completed this assessment (8% response (7/87) from the first 5% random sample, and 76% (139/181) from the second), within which one child was referred for a research diagnostic assessment (step 3), but this child did not meet research diagnostic criteria.

In the low-score group, 750 children were identified by teachers and school psychologists as showing concerns, and all received a clinical assessment (step 2), of whom three were referred for and completed a research diagnostic assessment (step 3), with one child meeting autism diagnostic criteria.

**Fig. 5:**
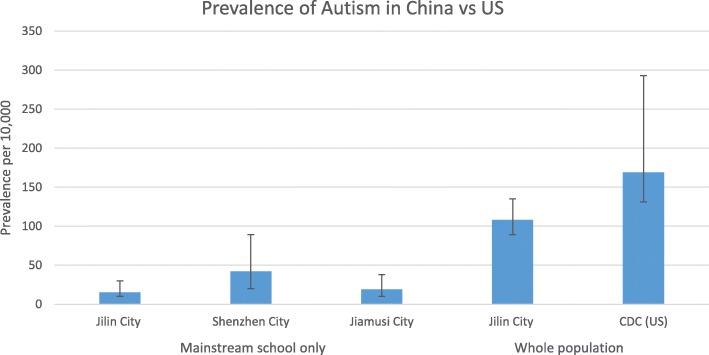
Prevalence of autism in China vs. USA

The 10 cases equate to a prevalence of 19.0 per 10,000 children age 6–10 (95% CI 9.7–37.5), adjusted for non-response. The prevalence for boys was 35.9 (95%CI 17.4–73.8) and girls 3.2 (95%CI 0.4–22.6) per 10,000. Mean IQ for the 10 children was 116. The large differences seen between Jiamusi and Shenzhen cities did not reach statistical significance either as a crude effect (OR = 0.45 95% CI 0.16–1.23, *p* = 0.12) or after adjusting for age, sex, income, and education differences between the two regions (OR = 0.39, 95% CI 0.12–1.30, *p* = 0.13). The results of the three cities are shown in Fig. 5 (with data from the mainstream schools and from the whole population), together with data from the recent CDC survey from the USA for comparison.

### Non-responders

In Shenzhen, non-response to the invitation for a clinical diagnosis was related to mother’s education (the higher the education the less likely the child was to take part, OR = 0.8, *p* = 0.016). Invited individuals in the borderline group were less likely to respond than those in the high group (OR = 0.6, *p* = 0.02). The following variables were not significantly related to non-response: father’s education, mother’s or father’s age or occupation, who completed the questionnaire (mother or father), income, being born in the city, age or sex of child (after adjusting for mother’s education), or CAST score (within groups). However, in Jiamusi, the patterns were slightly different: children were more likely to take part if they were older, and with response differences for both mother’s education and father’s education, and again with those less educated more likely to take part. Income was also inversely related to participation, with those on lowest incomes most likely to participate.

The following variables were not significantly related to response rate: mother’s or father’s age, who completed the questionnaire, or mother’s occupation. After adjusting for the other factors, father’s education and father’s occupation (higher social status occupations were less likely to take part), and child’s age were the strongest factors related to non-response. In addition, the higher the CAST score the less likely the child was to take part, though this effect was small. More detailed results are presented in Appendix 2 Detailed results’ tables.

## Discussion

### Key findings

There are four key findings from this study. First, results from Jilin City where both mainstream and special school data were available revealed a similar prevalence of autism in China to the West, at around 1% [24–27]. Second, in Shenzhen and Jiamusi cities, where only mainstream data were available, prevalence is also in line with Western estimates [9, 26]. Third, in all three cities, new cases of autism were identified by the study in mainstream schools, reflecting current under-diagnosis. Finally, non-significant variation across different cities is seen indicating the need to explore potential variation of autism across diverse Chinese regions with large sample sizes to achieve a fully robust national picture.

The prevalence estimate in mainstream schools in Jilin was 14.6 per 10,000. In Shenzhen, it was 42.3 per 10,000 and 19.0 per 10,000 in Jiamusi. In Jilin, where prevalence included those children with autism identified from special education and other settings outside mainstream population, the prevalence estimate was 108 per 10,000.

### Strengths

The strengths of this first large-scale study of autism in China are several. We used a total population approach covering all services in one city and mainstream schools in the other two. The response rate was high, much higher than has ever been achieved in the West. We used internationally agreed standardised screening and diagnostic instruments in all three cities. This included a Mandarin Chinese version of the CAST that has previously been shown to have acceptable validity in a Chinese population [21]. The Western-developed diagnostic instruments ADI-R and ADOS were used in combination with clinical diagnosis by Chinese child psychiatrists and were confirmed to be acceptable within a Chinese clinical setting, finding that these produce similar prevalence estimates across both Western and Asian cultures.

After screening (step 1), this study used local child psychiatrists as an additional selection step (step 2) prior to being followed up with research diagnostic assessments (step 3) which is a modification from previous prospective epidemiological studies in other countries, reducing the volume of false positives which otherwise may be experienced in such studies [3, 4, 6, 26]. The population size covered by the sampling in each of the three cities was large and included both screen-positives, screen-borderline, and screen-negatives in all three score groups for diagnostic assessments. Finally, our analytical methods took this staged study design into account, along with drop out between the stages, in our estimation of confidence intervals.

### Limitations

Despite these strengths, there are several limitations. First, according to the recruitment criteria, the sample for the cities was representative for the local region but is not yet nationally representative of China; hence, the hope for the China SCORE project is to map autism prevalence in 10 cities in total. It is important for national policy to report data from these first three cities, and our on-going data collection in the seven other cities will serve to further inform national policy and future research directions. The impact of differences in the three regions is difficult to establish given considerable variation in economic status and demographic characteristics for the three regions.

In Shenzhen and Jiamusi cities, not all of the schools took part in this study. Non-participation is not unusual in any epidemiological studies, particularly those addressing neuropsychiatric disorders. The influence of non-participation in epidemiological studies of autism has been discussed [28, 29], especially those with low participation rates. As participation has to be voluntary under ethical codes, the participation of each local government relies on an existing collaboration. It is not uncommon that some communities will not wish to take part. In addition, individual and family participation is subject to the usual human research ethical codes and participation is voluntary. The participation rate of the communities in this study is much higher than most of the prevalence studies of autism internationally. Although there will be non-participation effects, these will be much less marked than those experienced by other such studies.

The use of both DSM-IV-TR and DSM-5 may lead to concerns about the differences in prevalence across regions as differences between the DSM-IV-TR and the DSM-5 exist. The DSM-5 has not been fully adopted in most clinical settings in China; therefore, the DSM-IV-TR criteria were also used in this study. The reasons for this were two-fold. First, to ensure there was consistency among child psychiatrists across the regions, and second, to ensure comparability between existing diagnosed children and newly diagnosed children. The Chinese DSM-5 was also provided to each psychiatrist to assist the diagnostic process. Children who were suspected to have autism according to either DSM-IV-TR or DSM-5 criteria were invited for a research assessment using the ADOS and the ADI-R. Thus, the potential impact due to differences between the DSM-IV-TR and the DSM-5 in this study should be minimised.

Missed diagnoses by child psychiatrists during clinical assessments serve to lower prevalence estimates. In the current study, we reduced the likelihood of this happening by developing a diagnostic agreement test that could be used by all psychiatrists in all participating regions. Psychiatrists recommended by CDPF only joined the final clinical assessment team if s/he achieved a diagnostic agreement of 80% or above. Thus, the final prevalence estimate should not be compromised by changes in personnel between the clinical diagnosis and the research diagnosis.

There could be variations in the inclusion of children with autism in mainstream schools that may have influenced prevalence estimates across the three cities. To date, there has been no unified healthcare system and health insurance policy in Mainland China for autism [9]. The current education inclusion policy only recommends mainstream schools to include children with disabilities, but this is not mandatory [9, 30]. In three cities, the participation rates of local schools during the screening and diagnostic phases were different. This could be explained by a number of issues. First, recognition and awareness of autism within the mainstream population in the three cities may be different. Data from the non-responders suggested that in Shenzhen, which is a more advanced city where awareness and understanding about autism might be higher, the mothers educated to a higher level were more likely to participate. In contrast, in Jiamusi city where the economic status was relatively lower, awareness about autism might be lower, and therefore parents educated to a higher level and those with higher socioeconomic status were less likely to participate.

Second, involvement and support from the local government for this study was different in each city. Third, participation in the study is voluntary, so the willingness of the parents to participate may be different. The attitude of the parents towards incentives could be different. There have been studies focusing on the potential selection bias in prevalence studies of autism [28, 29]. Parental denial about autism may also contribute to differences in participation rates. It is possible that when parents realised that their child might have more difficulties than other children, they were less likely to participate. Thus, in both Shenzhen and Jiamusi cities, children who scored in the high score group were less likely to participate than those in the borderline score group. These factors might lead to a higher prevalence of autism in more advanced cities such as Shenzhen city and a lower prevalence in less developed cities such as Jiamusi city.

Another limitation of this study was that there was no information available concerning the prevalence of autism in special education in Shenzhen and Jiamusi cities. The goal of the study was to report the prevalence of autism in a previously neglected population, i.e., the mainstream school population. In China, most of the previously identified cases of autism are children with severe autism, including those with intellectual disability. To date, nearly all of the prevalence studies of autism in China have been carried out in special education settings but not in mainstream settings [8, 9]. In contrast to developed countries, most children with moderate to severe autism in China do not attend mainstream schools. They would not be admitted during the application process for kindergartens and primary schools [9, 30].

Children with autism who do not have intellectual disability are more likely be identified and given a diagnosis of autism in developed countries than in China [30, 31]. This is one of the reasons why previous prevalence estimates were much lower than those from the developed countries. In 2015, we reported our pilot study in two mainstream schools in Beijing which showed there were children with autism in mainstream schools. The prevalence in those two schools was 119 per 10,000 [14]. The current study was carried out in a large mainstream population in those three cities.

## Conclusions

The prevalence estimate indicates that the prevalence of autism in China is similar to estimates in Western countries. As China has such a large population, addressing this under-diagnosis of children with autism in mainstream schools would need major improvement in healthcare and education systems to support their families. Children with autism in primary schools are mostly children with average IQ (mean IQ > 100, Additional file 1). These children urgently need support to understand the difficulties and challenges they might face in the future to prepare themselves for school and the workplace.

During previous and current studies, we found there was a lack of awareness and knowledge among school professionals about autism [5, 10, 11]. Many schools do not have special education teachers. Many parents had never heard of autism prior to the study. If sufficient information is provided to parents at the hospital paediatric department, this could profoundly improve early detection of autism. Recognition of the role that child psychiatrists play throughout the life-course needs to be improved in China. Even among school psychologists, many of them had no awareness about symptoms of autism before we gave them training about screening and diagnosis. Special teachers and school psychologists need to be trained with skills and intervention strategies to help children who may be struggling because of social and communication difficulties that may be related to autism.

### Additional file

Additional file 1: Supplementary information. (DOCX 28 kb)

## Appendix 1

### Prevalence review tables

**Table 4:** Summary of prevalence studies of autism spectrum disorders in China (25 studies)

Year	First author	Region	Sample size	Area	Age	Sample screened	Screen methods	Screen tools	Cut-off	Response rate	P/R	Diagnostic tools	Diagnostic criteria	Childhood autism prevalence/SE (per 10,000)	ASC prevalence/SE (per 10,000)
1987	Tao [25]	Mainland	457,200	Urban	3–8	C	R	N/A	N/A	N/A	R	N/A	Rutter	0.32 (0.08)	–
2000	Luo [51]	Mainland	10,802	Mixed	2–14	SG	QI	ABC	31	100%	P	N/A	CCMD-2-R, DSM-III-R	2.8 (1.60)	–
2002	Wang [30]	Mainland	3.978	Urban	2–6	K	QI	CABS*	7	98.3%	P	CARS	CCMD-2-R	17.9 (6.70)	–
2002	Ren [43]	Mainland	3559	Urban	3–5	SG	QI	CABS	14	99.1%	P	N/A	N/A	250 (2.31)	–
2003	Wang[52]	Mainland	7488	Mixed	2–6	SG	QI	CABS	7	98.08%	P	CARS	CCMD-2-R	12.3 (4.05)	–
2003	Chang [26]	Taiwan	660	Mixed	15–93	C	C	ASDASQ	5	100%	P	N/A	DSM-IV	–	60.0 (30.06)
2004	Guo [53]	Mainland	5000	Urban	0–6	WP	QI	CABS	7	99.1%	P	CARS	CCMD-2-R	10 (4.47)	–
2004	Guo [54]	Mainland	3776	Rural	2–6	SG	QI	CABS	7	100%	P	CARS	DSM-IV	8 (4.59)	–
2005	Zhang [55]	Mainland	7416	Urban	2–6	SG	QI	CABS	7	99%	P	CARS	DSM-IV	11.0 (3.85)	–
2005	Zhang [29]	Mainland	1305	Urban	3–7	K	QI	CABS	14	100%	P	N/A	N/A	19.9 (2.47)	–
2005	Liu [56]	Mainland	21,866	Mixed	2–6	SG	QI	CABS	7	100%	P	CARS	DSM-IV	13.4 (2.47)	15.3 (2.64)
2007	Yang [31]	Mainland	10,412	Urban	3–12	PS	QI	ABC	31	100%	P	N/A	DSM-IV	5.6 (2.32)	–
2007	Wong [22]	Hong Kong	4,247,206	Mixed	0–14	HS	R	N/A	N/A	N/A	R	CARS, ADI-R	DSM-IV	–	16.1 (0.19)
2008	Zhang [21]	Mainland	8681	Urban	2–3	SG	QI	CHAT	N/A	100%	P	CARS	DSM-IV	16.1 (4.3)	–
2008	Zhang [21]	Mainland	12,430	Urban	4–6	SG	QI	CABS	14	100%	P	CARS	DSM-IV	8.85 (2.7)	–
2009	Zhang [57]	Mainland	5000	Urban	0–6	SG	QI	CABS	7	99.98%	P	CARS	CCMD-2-R	10.0 (4.47)	–
2009	Wang [28]	Mainland	4156	Urban	2–6	K	QI	CABS	14	100%	P	N/A	N/A	19.5 (6.84)	–
2010	Li [58]	Mainland	8006	Mixed	1.5–3	SG	QI	CHAT	N/A	92.99%	P	CARS	DSM-IV	26.2 (5.71)	–
2010	Wu [59]	Mainland	8532	Urban	0–3	SG	QI	CHAT	N/A	100%	P	CARS	DSM-IV	8.2 (3.10)	–
2010	Yu [33]	Mainland	7059	Mixed	2–6	SG	Q	CABS	7	89.7%	P	N/A	DSM-IV	21.2 (5.47)	22.7 (5.66)
2010	Chen [32]	Mainland	7034	Mixed	2–6	SG	Q	CABS	7	98.78%	P	CARS	DSM-IV	14.2 (4.49)	24.2 (5.86)
2011	Wang [27]	Mainland	7500	Urban	2–6	K	QI	CABS	14	87.8%	P	N/A	DSM-IV	29.5 (6.26)	75.4 (9.99)
2011	Liang [60]	Mainland	2485	Urban	3–6	K	QI	CABS	14	100%	P	N/A	DSM-IV, ICD-10	14.1 (7.53)	–
2011	Li [24]	Mainland	616,940	Mixed	0–17	SG	QI	ABC	N/A	N/A	P	N/A	ICD-10	2.38 (0.20)	–
2011	Chien [23]	Taiwan	372,642	Mixed	0–17	HS	R	N/A	N/A	N/A	R	N/A	ICD-9	–	28.7 (0.88)

–, data not available. Sample screened: *C* clinical patients, *SG* stratified general population, *K* kindergartens, *WP* whole population, *PS* primary schools, *HS* population in health system; screen methods: *R* records, *QI* questionnaire-based interview, *C* clinical referral, *Q* questionnaire distribution. *ABC* Autism Behaviour Checklist, *CABS* Clancy Autism Behavioural Scale, *ASDASQ* Autism Spectrum Disorder in Adults Screening Questionnaire, *M-CHAT* The Modified Checklist for Autism in Toddlers, *CARS* Childhood Autism Rating Scale, *P* perspective, *R* retrospective, *CAST* Childhood Autism Spectrum Test, *ADOS* Autism Diagnostic Observation Schedule, *ADI-R* Autism Diagnostic Interview-Revised, *DSM-IV-TR* Diagnostic and Statistic Manual of Mental Disorders, 4th Edition, Text Revision, *DSM-V* Diagnostic and Statistical Manual of Mental Disorders, 5th Edition, *ICD-10* International Classification of Disease-10th revision

**Table 5:** Summary of prevalence studies of autism spectrum disorders in Chinese populations between 2012 and 2016 (10 studies)

Year	First author	Region	Sample size	Area	Age	Sample screened	Screen methods	Screen tools	Response rate	P/R	Diagnostic tools	Diagnostic criteria	Childhood autism prevalence/SE (per 10,000)	ASC prevalence/SE (per 10,000)
2012	Uncertain	Mainland	148,030	Ningxia	0–14	WP	R	ABCS	–	R	CARS	ICD-10	5.2	–
2013	Lai	Taiwan	4,004,997	Taiwan	3–17	WP	R	–	–	R	–	–	–	
2014	Chen	Mainland	5500	Zhuhai	1.5–3	SG	Q	M-CHAT	90.9%	P	CARS	DSM-IV	–	29.5
2014	Deng	Mainland	4980	Henyang	3–6	K	Q	CABS	98.3%	P	CARS	DSM-IV	16.8	62.7
2015	Wang	Mainland	8000	Zaozhuang	2–6	K	Q	CABS	88.2%	P	CARS	DSM-V	–	66.3
2015	Jiang	Mainland	10,385	Shanghai	4–6	K	Q	CABS	93.1%	P	ADI-R	DSM-V	–	9.3
2015	Yang	Mainland	15,200	Shenzhen	4	K	Q	Q	91.2%	P	ABC	–	Uncertain (questionable autism 2.6%)	Uncertain
2015	Wang	Mainland	51,968	Shantou	3–6	WP	–	–	–	R	–	–	–	26.7
2015	Sun	Mainland	737	Beijing	6–10	WP	Q	CAST	97%	P	ADOS, ADI-R	DSM-IV	–	119
2016	Wang	Mainland	7463	Jilin	6–11	SG	Q	CAST	86.8%	P	–	CCMD-3	–	63.7

–, data not available. Sample screened: *C* clinical patients, *SG* stratified general population, *K* kindergartens, *WP* whole population, *PS* primary schools, *HS* population in health system; screen methods: *R* records, *QI* questionnaire-based interview, *C* clinical referral, *Q* questionnaire distribution. *ABC* Autism Behaviour Checklist, *CABS* Clancy Autism Behavioural Scale, *ASDASQ* Autism Spectrum Disorder in Adults Screening Questionnaire, *M-CHAT* The Modified Checklist for Autism in Toddlers, *CARS* Childhood Autism Rating Scale, *P* perspective; *R* retrospective, *CAST* Childhood Autism Spectrum Test, *ADOS* Autism Diagnostic Observation Schedule, *ADI-R* Autism Diagnostic Interview-Revised, *DSM-IV-TR* Diagnostic and Statistic Manual of Mental Disorders, 4th Edition, Text Revision, *DSM-V* Diagnostic and Statistical Manual of Mental Disorders, 5th Edition, *ICD-10* International Classification of Disease-10th revision

## Appendix 2

**Table 6:** Age and sex distribution of Shenzhen City sample

Age	Sex		Missing	Total (%)
	Boys	Girls		
6	2179	1879	7	4065 (19.0)
7	3237	2459	16	5712 (26.7)
8	3110	2387	20	5517 (25.8)
9	2454	1968	12	4434 (20.7)
10	632	420	2	1054 (4.9)
Others	266	199	173	638 (3.0)
Total	11,878	9312	230	21,420 (100)

**Table 7:** Characteristics of Shenzhen sample within age range during clinical assessments

Variables	*n* (%)	Group 1: CAST ≥ 15		Group 2: 12–14			Group 3: ≤ 11	
		Completed	Not completed	Invited and completed	Invited but not participated	Not invited	Invited	Not invited
Number		796 (67)	391 (33)	70 (3)	53 (2)	2419 (95)	23 (0.1)	17,050 (99.9)
CAST score	Median (IQR)	16 (15,17)	16 (15.17)	13 (12, 14)	13 (12, 14)	13 (12, 13)	13 (12, 14)	13 (12, 13)
	Mean (SD)	15.5 (3.7)	15.8 (3.0)	12.2 (2.5)	12.6 (1.7)	12.6 (1.6)	12.2 (2.5)	12.6 (1.6)
Age	Mean (SD)	8.2 (1.1)	8.3 (1.1)	7.9 (1.1)	8.4 (1.0)	8.2 (1.1)	7.9 (1.1)	8.2 (1.1)
Sex								
Boys		527 (66)	265 (68)	40 (57)	35 (66)	1548 (64)	22 (96)	9185 (54)
Girls		241 (30)	126 (32)	30 (43)	18 (34)	866 (36)	1 (4)	7840 (46)
Missing		28 (3.5)	0 (0)	0 (0)	0 (0)	5 (0.2)	0 (0)	25 (0.2)
Mother’s age								
≤ 24		5 (0.6)	1 (0.3)	0 (0)	0 (0)	11 (0.5)	0 (0.0)	44 (0.3)
25–30		88 (11.1)	47 (12.0)	8 (11.4)	5 (9.4)	337 (13.9)	4 (17.4)	2063 (12.1)
31–34		419 (52.6)	211 (54.0)	35 (50.0)	31 (58.5)	1330 (55.0)	12 (52.2)	9883 (58.0)
35–40		184 (23.1)	110 (28.1)	14 (20.0)	12 (22.6)	565 (23.4)	7 (30.4)	3924 (23.0)
≥ 41		19 (2.4)	2 (0.5)	3 (4.3)	1 (1.9)	40 (4.3)	0 (0.0)	345 (2.0)
Missing		81 (10.2)	20 (5.1)	10 (14.3)	4 (7.6)	136 (5.6)	0 (0.0)	791 (4.6)
Father’s age								
≤ 24		2 (0.3)	1 (0.3)	0 (0.0)	0 (0.0)	6 (0.3)	0 (0.0)	23 (0.1)
25–30		21 (2.6)	8 (2.1)	1 (1.4)	2 (3.8)	92 (3.8)	2 (8.7)	551 (3.2)
31–34		393 (49.4)	183 (46.8)	34 (48.6)	30 (56.6)	1215 (50.2)	9 (39.1)	8604 (50.5)
35–40		265 (33.3)	169 (43.2)	21 (30.0)	18 (34.0)	869 (37.0)	11 (47.8)	6197 (36.4)
≥ 41		45 (5.7)	12 (3.1)	5 (7.1)	2 (3.8)	85 (3.5)	1 (4.4)	835 (4.9)
Missing		70 (8.8)	18 (4.6)	9 (12.9)	1 (1.9)	125 (5.2)	0 (0.0)	840 (4.9)
Mother’s education								
Junior high or lower		366 (46.0)	168 (43.0)	35 (50.0)	18 (34.0)	1023 (42.29)	11 (47.8)	5359 (31.4)
High school		252 (31.7)	130 (33.3)	25 (35.7)	21 (39.6)	908 (37.5)	8 (34.8)	6972 (40.9)
College or university		116 (14.6)	71 (18.2)	5 (7.1)	12 (22.6)	416 (17.2)	4 (17.4)	4248 (24.9)
Graduate school		8 (1.0)	6 (1.5)	0 (0.0)	1 (1.9)	14 (0.6)	0 (0.0)	125 (0.7)
Missing		54 (6.8)	16 (4.1)	5 (7.1)	1 (1.9)	58 (2.4)	0 (0.0)	346 (2.0)
Father’s education								
Junior high or lower		264 (33.1)	115 (29.4)	22 (31.4)	16 (30.2)	729 (30.1)	10 (43.5)	3795 (22.3)
High school		285 (35.8)	144 (36.83)	30 (42.9)	1040 (40.9)	988 (40.8)	7 (30.4)	6732 (39.5)
College or university		175 (22.0)	101 (25.8)	14 (20.0)	627 (24.7)	602 (24.9)	4 (17.4)	5697 (33.4)
Graduate school		14 (1.8)	10 (2.6)	1 (1.4)	29 (1.1)	26 (1.1)	0 (0.0)	321 (1.9)
Missing		58 (7.3)	21 (5.4)	3 (4.3)	79 (3.1)	74 (3.1)	2 (8.7)	505 (3.0)
Mother’s occupation								
Public servant hired by the government		6 (0.8)	1 (0.3)	0 (0.0)	0 (0.0)	18 (0.7)	0 (0.0)	64 (0.4)
Company clerks		213 (26.8)	93 (23.8)	13 (18.6)	13 (18.6)	611 (25.3)	3 (13.0)	5226 (30.7)
Industry workers		45 (5.7)	28 (7.2)	6 (8.6)	6 (8.6)	157 (6.5)	2 (8.7)	1232 (7.2)
Self-employed		191 (24.0)	109 (27.9)	21 (30.0)	21 (30.0)	669 (27.7)	6 (26.1)	4451 (26.1)
Other worker		79 (9.9)	27 (6.9)	3 (4.3)	3 (4.3)	237 (9.8)	3 (13.0)	1224 (7.2)
Student		1 (0.1)	1 (0.3)	0 (0.0)	0 (0.0)	3 (0.1)	0 (0.0)	27 (0.2)
Farmer		49 (6.2)	28 (7.2)	11 (15.7)	11 (15.7)	158 (6.5)	2 (8.7)	684 (4.0)
Unemployed		206 (25.9)	103 (26.3)	16 (22.9)	16 (22.9)	558 (23.1)	7 (30.4)	4102 (24.1)
Missing		6 (0.8)	1 (0.3)	0 (0.0)	0 (0.0)	8 (0.3)	0 (0.0)	40 (0.2)
Father’s occupation								
Public servant hired by the government		8 (1.0)	5 (1.3)	0 (0.0)	0 (0.0)	35 (1.5)	1 (4.4)	244 (1.4)
Company clerks		236 (29.7)	108 (27.6)	23 (32.9)	14 (26.4)	741 (30.6)	11 (47.8)	6385 (37.5)
Industry workers		66 (8.3)	49 (12.5)	8 (11.4)	4 (7.6)	219 (9.1)	1 (4.4)	1489 (8.7)
Self-employed		272 (34.2)	148 (37.9)	25 (35.7)	24 (45.3)	900 (37.2)	6 (26.1)	6242 (36.6)
Other worker		95 (11.9)	34 (8.7)	3 (4.3)	6 (11.3)	262 (10.8)	3 (13.0)	1287 (7.6)
Student		2 (0.3)	0 (0.0)				0 (0.0)	8 (0.1)
Farmer		36 (4.5)	16 (4.1)	6 (8.6)	1 (1.9)	111 (4.6)	0 (0.0)	418 (2.5)
Unemployed		75 (9.4)	31 (7.9)	5 (7.1)	4 (7.6)	148 (6.1)	1 (4.4)	935 (5.5)
Missing		6 (0.8)	0 (0.0)	0 (0.0)	0 (0.0)	3 (0.1)	0 (0.0)	42 (0.3)
Income								
≤ 1999		46 (5.8)	31 (7.9)	4 (5.7)	4 (7.6)	135 (5.6)	0 (0.0)	609 (3.6)
2000–3999		181 (22.7)	89 (22.8)	17 (24.3)	14 (26.4)	480 (19.8)	5 (21.7)	2569 (15.1)
4000–5999		156 (19.6)	81 (20.7)	16 (22.9)	12 (22.6)	515 (21.3)	9 (39.1)	3047 (17.9)
6000–7999		104 (13.1)	38 (9.7)	10 (14.3)	7 (13.2)	336 (13.9)	3 (13.0)	2552 (15.0)
8000–9999		68 (8.5)	36 (9.2)	4 (5.7)	4 (7.6)	227 (9.4)	1 (4.4)	2049 (12.0)
≥ 10,000		112 (14.1)	69 (17.7)	10 (14.3)	6 (11.3)	500 (20.7)	3 (13.0)	4699 (27.6)
Missing		129 (16.2)	47 (12.0)	9 (12.9)	6 (11.3)	226 (9.3)	2 (8.7)	1525 (8.9)

**Table 8:** Age and sex distribution of Jiamusi City sample

Age	Sex		Missing	Total (%)
	Boys	Girls		
6	1918	1916	10	3844 (23.5)
7	1930	1730	11	3671 (22.4)
8	1952	1810	10	3772 (23.1)
9	1875	1908	6	3789 (23.2)
10	230	161	1	392(2.4)
Others	421	328	141	890 (5.4)
Total	8326	7853	179	16,358 (100)

**Table 9:** Characteristics of Jiamusi sample within age range during clinical assessments

Variables	*n* (%)	Group 1: CAST ≥ 15		Group 2: 12–14			Group 3: ≤ 11	
		Completed	Not completed	Invited and completed	Invited but not participated	Not invited	Invited	Not invited
Number		414 (46)	487 (54)	144 (8)	124 (7)	1554 (85)	750 (6)	12,190 (94)
CAST score	Median (IQR)	16 (15,17)	16 (15.17)	12 (12, 13)	13 (12, 14)	13 (12, 13)	7 (5,9)	7 (5, 9)
	Mean (SD)	16 (2.6)	16 (3.9)	12 (1.8)	12 (2.1)	12 (1.8)	7 (2.4)	6 (2.5)
Age	Mean (SD)	8.2 (1.3)	7.8 (1.7)	7.9 (1.5)	7.8 (1.3)	8.0 (1.3)	7.9 (1.1)	8.2 (1.1)
Sex								
Boys		260 (63)	337 (69)	80 (56)	63 (51)	921 (59)	376 (50)	5979 (49)
Girls		151 (36)	148 (30)	64 (44)	59 (48)	625 (40)	373 (48)	6189 (46)
Missing		3 (0.7)	2 (0.4)	0 (0)	2 (1.6)	8 (0.5)	1 (0.3)	22 (0.2)
Mother’s age								
≤ 24		3 (0.7)	2 (0.4)	0 (0)	0 (0)	4 (0.3)	2 (0.3)	27 (0.2)
25–30		64 (15.5)	84 (17.3)	28 (19.4)	16 (12.9)	237 (15.3)	149 (19.9)	1462 (12.0)
31–34		215 (51.9)	230 (47.2)	87 (60.4)	61 (49.2)	831 (53.5)	444 (59.2)	6811 (55.9)
35–40		37 (8.9)	31 (6.4)	10 (6.9)	7 (5.7)	112 (7.2)	50 (6.7)	713 (5.9)
≥ 41		0 (0)	0 (0)	0 (0)	0 (0)	0 (0)	0 (0.0)	2 (0.0)
Missing		95 (23.0)	140 (28.8)	19 (13.2)	40 (32.3)	370 (23.8)	105 (14.0)	3175 (26.1)
Father’s age								
≤ 24		2 (0.5)	3 (0.6)	0 (0.0)	0 (0.0)	6 (0.4)	1 (0.1)	14 (0.1)
25–30		32 (7.7)	37 (7.6)	12 (8.3)	12 (9.7)	108 (7.0)	70 (9.3)	532 (4.4)
31–34		262 (63.3)	280 (57.5)	105 (72.9)	75 (60.5)	937 (60.3)	534 (71.2)	8128 (66.7)
35–40		50 (12.1)	52 (10.7)	16 (11.1)	12 (9.7)	188 (12.1)	68 (9.1)	1223 (10.0)
≥ 41		0 (0.0)	0 (0.0)	0 (0.0)	0 (0.0)	0 (0.0)	0 (0.0)	2 (0.2)
Missing		68 (16.4)	115 (23.6)	11 (7.6)	25 (20.2)	315 (20.3)	77 (10.3)	2291 (18.8)
Mother’s education								
Junior high or lower		223 (53.9)	225 (46.2)	97 (67.4)	56 (45.2)	804 (51.7)	496 (66.1)	4583 (37.6)
High school		133 (32.1)	160 (32.9)	36 (25.0)	44 (35.5)	455 (29.3)	194 (25.9)	4258 (34.9)
College or university		37 (32.1)	79 (16.2)	4 (2.8)	20 (16.1)	231 (14.9)	48 (6.4)	2997 (24.6)
Graduate school		6 (1.5)	7 (1.4)	2 (1.4)	1 (0.8)	18 (1.2)	6 (0.8)	217 (1.8)
Missing		15 (3.6)	16 (3.3)	5 (3.5)	3 (2.4)	46 (3.0)	6 (0.8)	135 (1.1)
Father’s education								
Junior high or lower		220 (53.1)	209 (42.9)	99 (68.8)	51 (41.1)	743 (47.8)	492 (65.6)	4349 (35.7)
High school		124 (30.0)	152 (31.2)	36 (25.0)	52 (41.9)	506 (32.6)	197 (26.3)	4335 (35.6)
College or university		47 (11.4)	96 (19.7)	5 (3.5)	17 (13.7)	241 (15.5)	44 (5.9)	3047 (25.0)
Graduate school		6 (1.5)	10 (2.1)	2 (1.4)	2 (1.6)	20 (1.3)	8 (1.1)	264 (2.2)
Missing		17 (4.1)	20 (4.1)	2 (1.4)	2 (1.6)	44 (2.8)	9 (1.2)	195 (1.6)
Mother’s occupation								
Public servant hired by the government		15 (3.6)	18 (3.7)	0 (0.0)	4 (3.2)	34 (2.2)	10 (1.3)	363 (3.0)
Company clerks		19 (4.6)	40 (8.2)	8 (5.6)	10 (8.1)	144 (9.3)	66 (8.8)	1586 (13.0)
Industry workers		26 (6.3)	44 (9.0)	5 (3.5)	8 (6.5)	134 (8.6)	31 (4.1)	1578 (13.0)
Self-employed		103 (24.9)	141 (29.0)	32 (22.2)	48 (38.7)	460 (29.6)	188 (25.1)	3735 (30.6)
Other worker		53 (12.8)	44 (9.0)	26 (18.1)	8 (6.5)	156 (10.0)	82 (10.9)	996 (8.2)
Student		0 (0.0)	0 (0.0)	0 (0.0)	0 (0.0)	0 (0.0)	0 (0.0)	7 (0.1)
Farmer		99 (23.9)	80 (16.4)	27 (18.8)	24 (19.4)	270 (17.4)	156 (20.8)	1415 (11.6)
Unemployed		87 (21.0)	109 (22.4)	46 (31.9)	20 (16.1)	325 (20.9)	215 (28.7)	2355 (19.3)
Missing		12 (2.9)	11 (2.3)	0 (0.0)	2 (1.6)	31 (2.0)	2 (0.3)	155 (1.3)
Father’s occupation								
Public servant hired by the government		6 (1.5)	23 (4.7)	0 (0.0)	4 (3.2)	51 (3.3)	10 (1.3)	696 (5.7)
Company clerks		32 (7.7)	38 (7.8)	10 (6.9)	10 (8.1)	124 (8.0)	41 (5.5)	1385 (11.4)
Industry workers		22 (5.3)	52 (10.7)	8 (5.6)	7 (5.6)	138 (8.9)	29 (3.9)	1386 (11.4)
Self-employed		103 (24.9)	126 (25.9)	33 (22.9)	41 (33.1)	472 (30.4)	253 (33.7)	4016 (33.0)
Other worker		99 (23.9)	81 (16.6)	38 (26.4)	26 (21.0)	283 (18.2)	155 (20.7)	2084 (17.1)
Student		0 (0.0)	0 (0.0)	0 (0.0)	0 (0.0)	0 (0.0)	0 (0.0)	5 (0.0)
Farmer		97 (23.4)	76 (15.6)	35 (24.3)	20 (16.1)	274 (17.6)	156 (20.8)	1444 (11.9)
Unemployed		38 (9.2)	70 (14.4)	19 (13.2)	13 (10.5)	163 (10.5)	99 (13.2)	913 (7.5)
Missing		17 (4.1)	21 (4.3)	1 (0.7)	3 (2.4)	49 (3.2)	7 (0.9)	261 (2.1)
Income								
≤ 1999		128 (30.9)	141 (29.0)	40 (27.8)	34 (27.4)	346 (22.3)	191 (25.5)	1869 (15.3)
2000–3999		150 (36.2)	161 (33.1)	49 (34.0)	45 (36.3)	550 (35.4)	311 (41.5)	4035 (33.1)
4000–5999		62 (15.0)	79 (16.2)	26 (18.1)	23 (18.6)	318 (20.5)	147 (19.6)	3270 (26.8)
6000–7999		22 (5.3)	26 (5.3)	10 (6.9)	8 (6.5)	121 (7.8)	43 (5.7)	1493 (12.3)
8000–9999		7 (1.7)	19 (3.9)	1 (0.7)	2 (1.6)	44 (2.8)	17 (2.3)	485 (4.0)
≥ 10,000		10 (2.4)	19 (3.9)	3 (2.1)	7 (5.7)	51 (3.3)	7 (0.9)	478 (3.9)
Missing		35 (8.5)	42 (8.6)	15 (10.4)	5 (4.0)	124 (8.0)	34 (4.5)	560 (4.6)

## Abbreviations

ADI-R: Autism Diagnostic Interview-Revised
ADOS: Autism Diagnostic Observation Schedule
ANOVA: One-way analyses of variance
CAST: Childhood Autism Spectrum Test
CDPF: China Disabled Persons’ Federation
DSM-IV-TR: Diagnostic and Statistical Manual of Mental Disorders, 4th Edition, Text Revision
DSM-V: Diagnostic and Statistical Manual of Mental Disorders 5th edition
RPM: Raven Progress Matrix
SCORE: Social Communication Research and Epidemiology
