# Supplementary material for: Autism prevalence in China is comparable to Western prevalence
Source: Mol Autism. 2019 Feb 28;10:7. doi: 10.1186/s13229-018-0246-0 (PMC6394100; doi:10.1186/s13229-018-0246-0)
Supplement: Supplementary file 1 — Supplementary information. (DOCX 28 kb) [file 13229_2018_246_MOESM1_ESM.docx]

**Title: Autism prevalence in China is comparable to Western prevalence**

**Appendix 2: Supplementary information**

**Study 1: Jilin City**

**Methods**

***Clinical diagnosis***

Child psychiatrists from Peking University Hospital conducted the face-to-face clinical assessments with the child and his/her parents based on *Diagnostic and Statistical Manual Fourth Edition*^1^ in the studied district. All the clinical assessments were conducted at schools where the children attended to encourage participation and to minimise the travel burden for participating families.

***Research diagnosis***

The Taiwanese versions of the ADOS and ADI-R (World Psychological Service, WPS) were used. The examiners who conducted the research diagnosis were trained prior to the start of the study and were blind to the outcome of the clinical assessments in order to avoid bias in the final consensus diagnosis. Prior to each assessment, parents were asked to provide consent for the assessment to take place and for the ADOS to be video-recorded and the ADI-R to be tape-recorded. The final research diagnoses were made following consensus diagnostic discussions with the child psychiatrists. Following the diagnostic assessment, a summary report was provided to each family giving general feedback about the child and a general summary report was given to primary schools. When the researcher had concerns about a child’s development a recommendation was made at the end of the summary report to parents.

***Data analysis***

A sensitivity analysis was conducted to investigate the effect of missing data on the CAST^2,3^ by re-running the analysis using the maximum score. If by using the maximum score, a change of score led to a change in the score group (from <12 to ≥12, or <15 to ≥15), the analyses were re-run without those individuals who changed score group to examine the stability of the results.

**Results**

None of the students had a previous diagnosis of autism. Of the 6,484 questionnaires, 6,149 (94.8%) were available for analysis. 335 5.2%）questionnaires were excluded as they had a name missing or more than 10 CAST items missing. In parallel, the number of children aged 6 and 10 years old who had an existing autism diagnosis not attending mainstream schools was 91. All 91 questionnaires were available for analysis. Thus, the total sample for analysis was 6,240 for the whole district.

In the non-mainstream school settings, 91 children were screened, 72 (79.1%) were in the high score group, 12 (13.2%) were in the borderline group and 7 (7.7%) were in the low score group. None of these children were invited for a further assessment as long as they provided a copy of their diagnostic report. If this was not provided but the child scored in the high score group, they were invited for a clinical diagnosis.

**Study 2: Shenzhen city**

**Methods**

***Population sample***

The sampling strategy was modified on the basis of the first city results. All the children who were in the high score group (CAST ≥ 15) were invited for a further assessment. Due to resource limitations, we aimed to assess all screen-positives. Therefore, not all children in the borderline group could be assessed.

***Diagnostic reliability***

An introductory training about autism and the screening approach was provided to school teachers and psychologists before the distribution of screening questionnaires at participating schools.

Most of the clinical assessments were conducted at school, with a minority of them conducted at hospital or local CDPF affiliated rehabilitation centres at the weekends if there were families that could not complete or attend assessments during weekdays at school. Assessments were conducted after obtaining consent from the parent. Clinical and research assessments were recorded to ensure the quality of the assessment and for reliability checking during the assessment phase.

For children who received a non-autism clinical diagnosis, a random sample was selected and invited for a research diagnostic assessment to confirm whether there were possible cases of autism being missed during the clinical assessment process. The number of children who received a non-autism diagnosis who were selected for a research assessment was determined by the following ratio: 10 autism/suspected autism children for every 1 non-autism child. The general coordinator was in charge of assigning children and parents for assessments, so the examiners were blind to the clinical status of the children.

The reliability between ADOS and ADI-R examiners was examined before the further assessment phase. Both ADOS/ADI-R examiners coded the same assessments and agreement was calculated using simple agreement of all scorable items on the two instruments. The agreement between ADOS examiners was 82%, and the reliability between ADI-R examiners was 93%. For each child, the ADOS and the ADI-R were conducted by different examiners to minimize the bias from the previous assessment.

***Case ascertainment***

A child was given a diagnosis of autism if s/he received a clinical diagnosis of autism, and met the cut-off for autism on the diagnostic algorithms on the ADOS and the ADI-R. Those children with any conflicting diagnostic outcome on the instruments or between the clinical and research diagnostic assessments, were reviewed by examining all materials and discussion between the clinicians and research examiners. Additional information was obtained from the school teachers if necessary. After discussion, if there were still difficulties in making a diagnosis, an external experienced child psychiatrist consultant was invited to review all the recorded materials and help with making a final consensus diagnosis. The consensus diagnosis was the final diagnosis. In addition, the IQ of child in research assessment was assessed using the Raven’s Progressive Matrices (RPM)^4,5^.

Following diagnostic assessment, a summary report was provided to each family, which gave general feedback about the child and a general summary report was given to the headteacher at school. At the end of assessment, the researcher had a 10 to 15 minutes conversation with the parent to help identify and answer any questions from parent about the development and behaviour of the child. If there were needs highlighted by the parent for further referral, another appointment with local child psychiatrists was arranged for the families. When the diagnostic team had concerns about a child’s development, a recommendation was made at the end of the summary report to parents. Contact information for enquiry at local CDPF affiliated centres was provided for such families in the invitation letter and consent form in the screening pack. Where families contacted the local CDPF centres, a further appointment with local child psychiatrists was arranged to provide a consultation about their child’s development and behaviours.

***Data analysis***

The 5% randomized sampling for the borderline group was conducted using STATA 14.0 with the total sample regardless of age range. The randomization of clinically diagnosed non-autism children was conducted using a random number table. This was conducted towards the end of the assessments to have an estimate of how many children in total would be clinically identified as having autism or suspected- autism. Then according to the ratio (10:1), the children who were non-autism were randomly selected.

**Results**

***Characteristics of sample***

The children who were outside the age range (6-10) were not excluded in the sampling for further assessment as we wanted to ask parents about the age when they came for assessment for children who scored high on the CAST. However, after further assessment, the analysis was based on children who were between 6 and 10 for the prevalence estimate. The mean age of the sample was 8.1 years old (SD=1.1). The distribution of age and sex is shown in Table 1. Of 20,802 children, 10,360 (49.8%) were originally born in Shenzhen, while 9,894 (47.6%) children migrated from other cities.

[insert Table 1 from Appendix 3]

A minority of CAST questionnaires were filled in by fathers (6,469, 31.1%), while 13,740 (66.1%) were filled in by mothers. The rest were completed by other caregivers. Occupation and education status of the parents were collected following the status of national population consensus from the national Bureau of Statistics^6^. Quality control using double data entry was conducted in 1% of returned questionnaires (n=215). Agreement between the first data entry and the second data entry was 98.6% (n=212). After the completion of data entry, a random selected 215 (1%) questionnaires were re-checked and the agreement on all items between the entered data and the original data was 99.5% (n=214).

***Distribution of Cast scores***

Within the study age range, using the minimum score of each child (all missing value as 0), the median score on the CAST was 7.8 (interquartile range: 5, 10; range: 0, 26; SD= 3.7). The distribution was positively skewed (skewness-kurtosis test: p<0.001). After handling missing values, the screened sample was distributed into three groups using the middle score. Of all the 21,420 children screened, 1,247 (5.8%) were in the high-score group (≥15), 2,663 (12.4%) were in borderline group (12-14), and most in the low-score group (17,510, 81.8%). This distribution was similar to a previous small sample study in Beijing using the CAST^7^. During screening, three children were reported to have an existing diagnosis of ASC by their parents.

***Diagnostic assessments***

The sampling for clinical assessments was made regardless the age of child as we wanted to capture all possible cases in this population and the missing value on age could be obtained from clinical assessment. Thus, using the total sample, all the children and their parents in the high-score group (N=1,247) were invited for a further assessment through the headteachers and schools. Within the research assessment sample, 75 children completed the Raven Progressive Matrix (RPM)^3^. The mean IQ was 104 (SD=16.8) and the median IQ was 106 (IQR: 95, 114; range: 58, 143). The children who received a consensus diagnosis of ASC had a mean IQ at 101 (SD=21.4).

***Analysis of non-participation***

There was no significant difference in the score distribution between children who participated in clinical assessment and those who did not participate (p=0.968); 3) There was no differences in medians of these two group (median test, p=0.509; Mann-Whitney test, p=0.4384).

In the high-score group, father’s occupation level was significantly different between who participated in clinical assessments and who did not (Chi-square -test=15.6, p=0.029). In the borderline group, the mother’s education of children who participated was significantly lower than those who did not participate (Kruskal-Wallis test, p=0.0284). The income of children who were not invited in borderline group was significantly higher than those who were invited for diagnosis (Kruskal-Wallis test, p=0.0469). In the low-score group, there were more boys than girls who completed the clinical assessments (Chi-square test, p<0.001). Fathers’ education level was lower for children who participated in the diagnosis (Mann-Whitney test, p=0.0128). The monthly income of families who participated in the clinical assessment in the low-score group was lower than those who did not participate (Mann-Whitney test, p=0.0496). The characteristics of children and their parents within different participation groups during clinical assessments are in Table 2.

[insert Table 2 from Appendix 3]

In the high-score group, the minimum score was significantly higher in children who were diagnosed or suspected as ASC by clinicians (Mann-Whitney test, p=0.0243). There were more boys in children who diagnosed by clinicians than those diagnosed as non-ASC (Chi-square test, p<0.001). The father’s age of children who were in research assessments were older than those were not invited to research assessments (Mann-Whitney test, p=0.0287).

***Agreement of assessment***

The research assessments of 12 children from Shenzhen assessment sample (10%, N=122) were re-examined by different examiners. Consensus agreement was for reached for only one child. The agreement was 91.7% among examiners.

**Study 3: Jiamusi city**

**Results**

***Quality of data***

Using all completed CAST questionnaire, the mean score was 7.9 (SD=3.7) and the median score was 7 (IQR: 5, 10; range: 0, 30). Quality control using double data entry was conducted in 1% of returned questionnaires (n=164). Agreement between the first data entry and the second data entry was 97.6% (n=160). After the completion of data entry, a random selected 164(1%) questionnaires were re-checked and the agreement on all items between the entered data and the original data was 97.0% (n=159). The distribution of age and sex of Jiamusi city is in Table 3.

[insert Table 3 from Appendix 3]

***Clinical assessment***

One child was given a diagnosis of Developmental Disability (DD) and 3 children were given a diagnosis of Intellectual disability (ID) during the clinical diagnosis.

***Research diagnosis***

According to the sampling rule, 3 children from the randomly selected non-ASC clinical category completed further assessments, of which none received an ASC diagnosis. Within the research assessment sample, 21 children completed the RPM. The mean IQ was 104 (SD=15.6) and the median IQ was 105 (IQR: 93, 1145; range: 75, 141). The children who received a consensus diagnosis of ASC had a mean IQ at 109 (SD=11.1).

***Analysis of non-participation***

During clinical assessment, in the high-score group, the age of children who participated in the clinical assessments was significantly older than children who did not participate (Mann-Whitney test, p=0.0063). In the borderline group, the minimum scores of children who participated in the clinical assessment were significantly lower than children who did not attend. On the contrary, in the low score group, the children who participated had a significantly higher score than those who were not assessed. Both parents’ education level was significantly lower in children who participated in the clinical assessments than those who did not participate in all three score groups. In all three groups, mother’s occupation was significantly different between children who participated and who did not. In the low score group, both parents’ age was significantly younger in children who participated. Father’s occupation was significantly different between children who participated and children who did not (Chi-square test, p<0.001). The income of the low-score group was significantly lower in children who participated (Mann-Whitney test, p<0.001).

In the second step, in the high-score group, there were significantly more boys who attended research diagnosis (Chi-square test, p=0.015). Both parents education level of children who attended the research diagnosis were higher than those who were not invited (Mann-Whitney test, p<0.01). Father’s occupation in the high-score group was significantly different between children who were invited for research assessment than those who were not invited (Mann-Whitney test, p<0.001). In the borderline group, the father’s age of children who attended the research assessments was older than those were not invited (Mann-Whitney test, p=0.0321).

***Reliability of diagnostic assessment***

For the clinical assessments, all the clinicians achieved diagnostic agreement prior to the clinical assessments. In Jiamusi study, 3 children (14%, N=22) were re-examined by different examiners and the agreement was 100%. Based on the sampling rule for non-ASC, 3 randomly selected children who were diagnosed as non-ASC by clinicians were also invited to research assessment and none of them met the diagnostic cut-off of ASC on the ADOS nor the autism cut-off on the ADI-R. The characteristics of children and their parents in different participation groups in clinical assessments are in Table 4.

[insert Table 4 from Appendix 3]

**References**

1. American Psychiatric Association. Diagnostic and Statistical Manual of Mental Disorders.(DSM-IV) (4th ed.). Washington DC: APA: American Psychiatric Association; 1994.
2. Williams J, Scott F, Stott C, et al. The CAST (Childhood Asperger Syndrome Test): test accuracy. *Autism* 2005; **9**(1): 45-68.
3. Baron-Cohen S, Scott FJ, Allison C, et al. Prevalence of autism-spectrum conditions: UK school-based population study. *The British Journal of Psychiatry* 2009; 194(6): 500-9.
4. Raven J, Raven JC, Court JH. Raven manual: standard progressive matrices. Oxford,UK: Oxford Psychologists Press; 1998.
5. Li D. Chinese Raven Test. Shanghai: East China Normal University Press; 1989.
6. National Bureau of Statistics of China. The national statistics on population. 2011 2012. <http://www.stats.gov.cn/>.
7. Sun X, Allison C, Matthews FE, et al. Exploring the Underdiagnosis and Prevalence of Autism Spectrum Conditions in Beijing. *Autism Res* 2015; **8**(3): 250-60.
